# A molecular computational model improves the preoperative diagnosis of thyroid nodules

**DOI:** 10.1186/1471-2407-12-396

**Published:** 2012-09-07

**Authors:** Sara Tomei, Ivo Marchetti, Katia Zavaglia, Francesca Lessi, Alessandro Apollo, Paolo Aretini, Giancarlo Di Coscio, Generoso Bevilacqua, Chiara Mazzanti

**Affiliations:** 1 Division of Surgical, Molecular, and Ultrastructural Pathology, Section of Molecular Pathology, University of Pisa and Pisa University Hospital, Via Roma 57, Pisa, 56100, Italy; 2 Section of Cytopathology, University of Pisa and Pisa University Hospital, Via Roma 57, Pisa, 56100, Italy

**Keywords:** Thyroid, Fine-needle aspiration (FNA), Area under the curve (AUC), Computational model, Preoperative diagnosis

## Abstract

**Background:**

Thyroid nodules with indeterminate cytological features on fine needle aspiration (FNA) cytology have a 20% risk of thyroid cancer. The aim of the current study was to determine the diagnostic utility of an 8-gene assay to distinguish benign from malignant thyroid neoplasm.

**Methods:**

The mRNA expression level of 9 genes (KIT, SYNGR2, C21orf4, Hs.296031, DDI2, CDH1, LSM7, TC1, NATH) was analysed by quantitative PCR (q-PCR) in 93 FNA cytological samples. To evaluate the diagnostic utility of all the genes analysed, we assessed the area under the curve (AUC) for each gene individually and in combination. BRAF exon 15 status was determined by pyrosequencing. An 8-gene computational model (Neural Network Bayesian Classifier) was built and a multiple-variable analysis was then performed to assess the correlation between the markers.

**Results:**

The AUC for each significant marker ranged between 0.625 and 0.900, thus all the significant markers, alone and in combination, can be used to distinguish between malignant and benign FNA samples. The classifier made up of KIT, CDH1, LSM7, C21orf4, DDI2, TC1, Hs.296031 and BRAF had a predictive power of 88.8%. It proved to be useful for risk stratification of the most critical cytological group of the indeterminate lesions for which there is the greatest need of accurate diagnostic markers.

**Conclusion:**

The genetic classification obtained with this model is highly accurate at differentiating malignant from benign thyroid lesions and might be a useful adjunct in the preoperative management of patients with thyroid nodules.

## Background

Thyroid nodules represent a very common problem. The majority (>95%) of them are benign; however, malignancy risk increases with female gender, nodule size, extremes of age (<30 and >60 years), personal or family history of thyroid malignancy and radiation exposure [1].

The advent of thyroid ultrasound allowed for an increasing number of nodules to be diagnosed, and it is now recognized that nodules are present in an estimated 50% of the general population and are detected at subclinical level. However, since only 10% of these nodules will be a true malignancy, preoperative testing to differentiate benign from malignant nodules are needed [2,3].

Currently, fine-needle aspiration (FNA) cytology is the most accurate and cost effective diagnostic test to exclude a thyroid cancer diagnosis. In general, a thyroid nodule on FNA cytology can be classified as benign, malignant, suspicious, indeterminate, or non-diagnostic [4].

Unfortunately, about 30% of FNAs are indeterminate and often require a diagnostic thyroidectomy to establish the diagnosis on permanent histological examination. Only 20% of diagnostic thyroidectomies in patients with indeterminate FNA cytology demonstrates malignant lesions on permanent histology, and these patients often require a completion thyroidectomy [5].

Therefore, because of this obvious limitation of FNA cytology in the preoperative diagnosis, there is a clinical need for reliable preoperative molecular markers to distinguish benign from malignant thyroid nodules.

A 10-gene (KIT, SYNGR2, C21orf4, Hs.296031, Hs.24183, FAM13A1, C11orf8, KIAA1128, IMPACT, CDH1) and a 6-gene (KIT, LSM7, SYNGR2, C21orf4, Hs.296031, Hs.24183) assays have been proposed to have high diagnostic accuracy to distinguish thyroid nodules [6]. Those assays have been developed from microarray analyses of tumor specimens obtained after surgical removal of thyroid nodules. Since only 20% of patients undergoing surgery have malignant lesions, preoperative tests are needed to avoid unnecessary surgery. Gene expression profiling studies have identified many other possible markers with high accuracy, however the clinical application of these markers is limited to the use of post-surgical samples. FNA cytology represents a useful tool in the preoperative evaluation of a thyroid nodule, especially because of the knowledge of the amount of tumor cells *per* sample. In a previous paper [7] we showed the clinical relevance of KIT expression to the diagnosis of thyroid tumors, whose RNA was extracted from cytological preoperative FNA specimens. Although KIT expression resulted to increase the diagnostic accuracy of 15% compared to the cytology alone, there were samples still remained indeterminate.

The aim of the current study was to build a q-PCR-based computational model able to preoperatively diagnose benign and malignant thyroid tumors on the basis of the expression profiles of the genes mentioned above (KIT, SYNGR2, C21orf4, Hs.296031, Hs.24183, CDH1, LSM7), plus two other genes (TC1, NATH) known to be involved in thyroid carcinogenesis from the literature [8-10]. In addition, since BRAF sequencing is so far the best molecular test used in the preoperative assessment of thyroid nodules malignancy, we also built a model including BRAF mutational status.

In the last years, a new class of techniques known as Bayesian Neural Networks (BNN) have been proposed as a supplement or alternative to standard statistical techniques. For the purpose of predicting medical outcomes, a BNN can be considered a computer intensive classification method and, in addition, BNNs do not require explicit distributional assumption (such as normality) [11].

As previously described by us, KIT is down-regulated in malignant thyroid tumors compared to the benign ones. SYNGR2 has been characterized as an integral vesicle membrane protein [12] and the only data available indicate its up-regulation in fetal mouse ovaries [13]. LSM7 has been described in the family of Sm-like proteins, involved in pre-messenger RNA splicing and decapping [14]. The interaction of LSM7 with the TACC1 complex may participate in breast cancer oncogenesis [15]. C21orf4 encodes a predicted trans-membrane protein (Tmem50b) and is one of few genes significantly over-expressed during cerebellar development in a Down syndrome mouse model [16]. The role of SYNGR2, LSM7 and C21orf4 in thyroid carcinogenesis has not yet been explored. E-Cadherin (CDH1) expression is reduced in thyroid carcinomas [17] and its promoter resulted to be hypermethylated in thyroid neoplasm [18]. Hs.24183 (now Hs.145049) has been identified as part of the 3’UTR of DDI2 (DNA-damage inducible 1 homolog 2) gene in H. sapiens, but no data exists about its role in thyroid. For Hs.296031 the only information available refers to gene sequence and mapping, but no gene and protein function are known yet. In contrast, the expression of the thyroid cancer-1 (TC1) gene resulted to be related to malignant transformation in thyroid and the potential use of TC1 gene expression as a marker of malignancy has also been shown in literature [19]. NATH (N-acetyl transferase human) is involved in protein acetylation which represents an important post-translational modification regulating oncogenesis, apoptosis and cell cycle. NATH resulted to be over-expressed at the mRNA level in papillary thyroid carcinomas compared to non-neoplastic thyroid tissue [8].

In this study we used 87 FNA cytological samples to build several preoperative computational models and 6 unknown samples to test in order to find the most discriminative one.

A correlation analysis between the markers was also performed in order to investigate their biological importance and to find a link that could give us a better understanding of the molecular mechanisms underlying thyroid cancer development.

## Methods

### Thyroid specimens

Preoperative thyroid FNA slides of a total of 93 patients carrying thyroid lesions (49 malignant, 38 benign, 6 unknown) were selected from archived materials of the Section of Cytopathology, Division of Surgical, Molecular and Ultrastructural Pathology, S. Chiara Hospital, Pisa. For ethical reasons we used only cases with two or more slides per patient and the molecular analysis was performed on only one of the available smears. In all cases FNA was performed using ultrasonography guidance. All smears were reviewed by a senior cytopathologist. Diagnosis was carried out on the basis of the following criteria broadly suggested in the literature: smear background, cell arrangements, cell shape, nuclear/cytoplasmic features, presence of nucleoli and mitosis. The histological diagnosis assessed ultimately the malignancy or benignity of the 93 thyroid lesions.

### Ethical board

This study was approved by the Internal Review Board of the University of Pisa. All patients gave their consent for the participation to the study.

### RNA and DNA isolation

Archival FNA slides stained with Papanicolaou technique were kept in xylene for 1 to 3 days, depending on the time of storage, in order to detach slide coverslips. The slides were then hydrated in a graded series of ethanol followed by a wash in distilled H_2_O for 1 minute. The slides were finally air dried. RNA extraction was performed using a commercial kit (High Pure RNA Paraffin kit, Roche). The lysis solution was poured on the slide to scrape off the cytological stained sample. Whole scraped tissue was then collected in a microcentrifuge tube and processed for RNA extraction. The quantity/quality of RNA was estimated with Nanodrop 1000 spectrophotometer using 1 μl of undiluted RNA solution. RNA was treated with DNase Ι recombinant, RNase-free (Roche). RNA was reverse transcribed in a final volume of 20 μl, containing 5X RT buffer, 10 mM dNTPs, 50 ng/μl Random Primers, 0.1 M DTT, 40 U/μl RNaseOUT, 50 μM oligo(dT), DEPC-Treated Water, 15 U/μl Cloned AMV reverse transcriptase (Invitrogen, Carlsbad, CA).

DNA was isolated directly from stained cells using a commercial kit (Nucleospin, Macherey-Nagel, Düren, Germany) according to the manufacturer’s instructions.

### Gene expression analysis

The level of KIT, SYNGR2, C21orf4, Hs.296031, DDI2, CDH1, LSM7, TC1, NATH expression was analysed by quantitative PCR (q-PCR) on the Rotor-Gene 6000 real time rotary analyzer (Corbett, Life Science, Australia) following the manufacturing instructions. Endogenous reference gene (B2M*,* beta 2 microglobulin) was used to normalize each gene expression level. PCR products were previously sequenced using the Applied Biosystems 3130xl Genetic Analyzer (Foster City, CA) to confirm gene sequence. PCR was performed in 25 μl final volume, containing 5 μl of cDNA, 12.5 μl of MESA GREEN qRT-PCR MasterMix Plus (EUROGENTEC, San Diego, CA), 40 pmol of each primer (Invitrogen, Carlsbad, CA) *per* reaction with the following cycling conditions: initial denaturation 95°C for 5 min; 40 cycles at 95°C for 15 sec, 61°C for 40 sec, 72°C for 40 sec; final step 25°C for 1 min. Primers were selected using Primer3 software:

KIT F: 5’- GCACCTGCTGCTGAAATGTATGACATAAT - 3’

KIT R: 5’- TTTGCTAAGTTGGAGTAAATATGATTGG - 3’

SYNGR2 F: 5’- ATCTTCTCCTGGGGTGTGCT - 3’

SYNGR2 R: 5’- AGGGTGGCTGTTGGTAGTTG - 3’

C21orf4 F: 5’- GACAACAGTGGCTGTGTTTTAAG - 3’

C21orf4 R: 5’- GCATTGGATACAGCATTTATCAT - 3’

Hs.296031 F: 5’- TGCCAAGGAGCTTTATAGAA - 3’

Hs.296031 R: 5’- ATGACGGCATGTACCAACCA - 3’

DDI2 F: 5’- TGCAGTTCCCAAACTTACCC- 3’

DDI2 R: 5’- CAGCAACATATCTCGGAGCA- 3’

CDH1 F: 5’- GCATTGCCACATACACTCTC- 3’

CDH1 R: 5’- AGCACCTTCCATGACAGAC- 3’

LSM7 F: 5’-GACGATCCGGGTAAAGTTCCA - 3’

LSM7 R: 5’- AGGTTGAGGAGTGGGTCGAA - 3’

TC1 F: 5’- AAATCTTCTGACTAATGCTAAAACG - 3’

TC1 R: 5’- TTATTGTTGCATGACATTTGC - 3’

NATH F: 5’-AAGAAACCAAAGGGGAACTT - 3’

NATH R: 5’- TAATAGGCCCAGTTTTCAGG - 3’

B2M F: 5’- CATTCCTGAAGCTGACAGCATTC - 3’

B2M R: 5’- TGCTGGATGACGTGAGTAAACC - 3’

Standard curves were generated for each gene for data analysis. To verify primers specificities, melting curve analysis was performed. Fluorescent data were acquired during the extension phase. After 40 cycles, a melting curve for each gene was generated by increasing the temperature from 50°C to 99°C (1°C for each step), while the fluorescence was measured. For each experiment a no-template reaction was included as a negative control.

For each cDNA sample the ratio between the expression value of the gene of interest and the expression value of B2M was calculated. Mean values and standard deviations of malignant and benign groups were calculated as well.

### BRAF status

BRAF sequence was screened for V600E mutation by pyrosequencing. DNA was first amplified using “Rotor-Gene 6000” (Corbett Research) and then sequenced using PyroMark Q96 ID system.

PCR was performed with the following conditions: initial denaturation 95°C for 3 min; 40 cycles at 95°C for 30 sec, 55°C for 30 sec, 72°C for 30 sec; final step 60°C for 5 min with TaKaRa Ex Taq (Qiagen). PCR amplification and mutational analysis were performed in accordance to the Diatech manual (Anti-EGFR MoAb response BRAF status).

### Statistical analysis

#### Gene expression analysis

Mann–Whitney test and Student’s *t*-test were used to determine differences between mRNA expression levels of KIT, LSM7, C21orf4, DDI2, SYNGR2, TC1, Hs.296031 and CDH1 and NATH, respectively. All the analyses were performed using Statgraphics Centurion (V. 15, StatPoint, Inc.).

#### ROC analysis

To determine the diagnostic accuracy of the molecular computational model, we calculated the area under the curve (AUC) of the receiver operating characteristic (ROC) curve for each gene individually and in combination using logistic regression analysis (Medcalc 11, Medcalc Software, Stata Software).

#### BNN classifier

Several computational models (Neural Network Bayesian Classifiers) were built in order to find the best combination of markers able to discriminate benign from malignant thyroid samples using Statgraphics Centurion (V. 15, StatPoint, Inc.).

#### Molecular diagnosis

Fisher’s test was used to compare samples correctly classified by the BNN model according to their probability score (> 90% and <90%). The diagnostic gain was then calculated after applying molecular tests (BRAF, KIT and BNN model).

#### Correlation analysis

In order to evaluate the biological importance of the markers analysed, a multiple-variable correlation analysis was performed between the markers (Partek software).

## Results

### Gene expression levels

**Figure 1:**
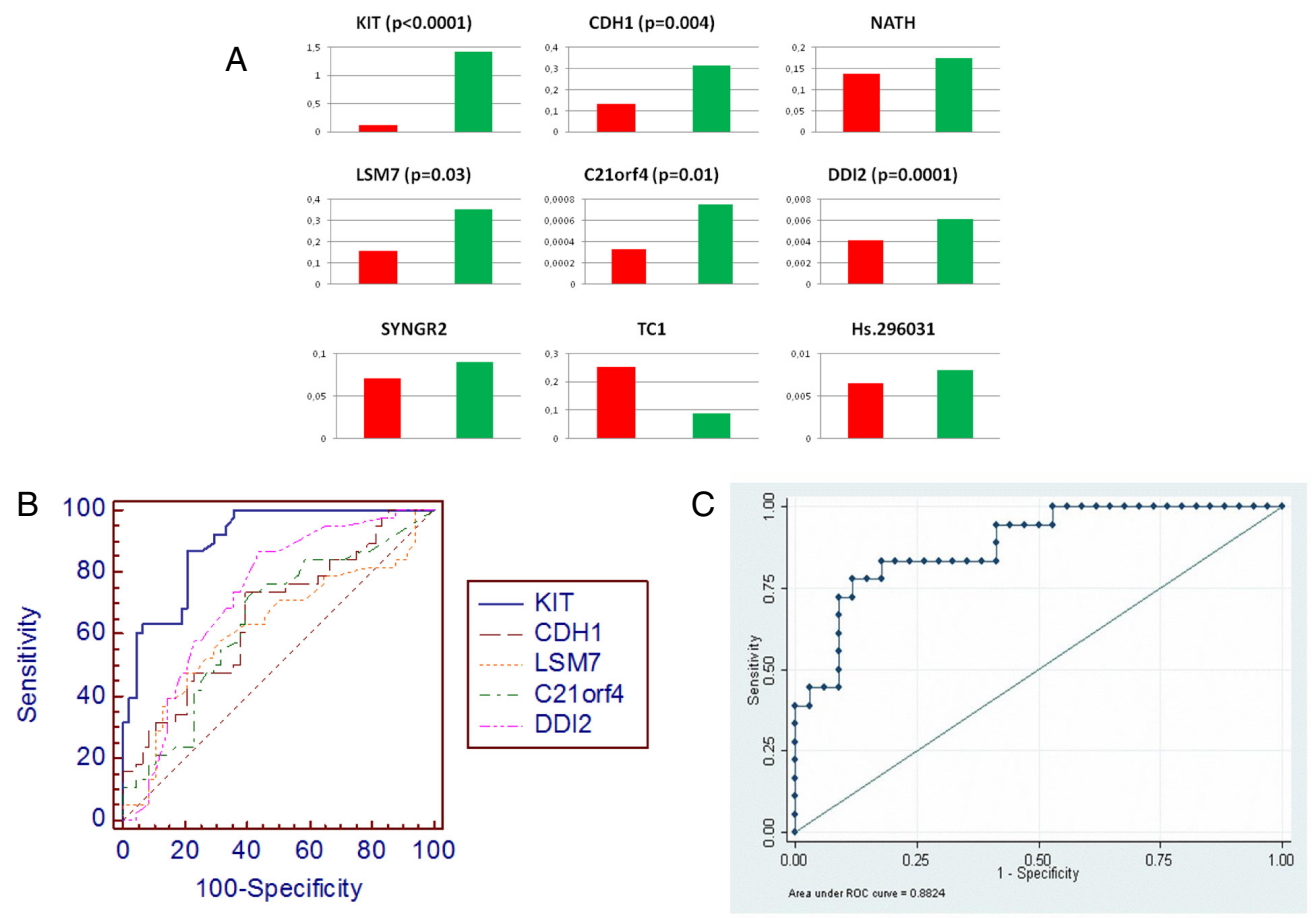
**Expression mean of 49 malignant (red) and 38 benign (green) samples for each marker (A).** ROC analysis for KIT, CDH1, LSM7, C21orf4, DDI2 separately. Among the markers, KIT resulted to be the most powerful in discriminating benign from malignant thyroid tumors (AUC = 0.9) (**B**). ROC analysis for KIT, CDH1, LSM7, C21orf4, DDI2, and BRAF status in combination (AUC = 0.88) (**C**).

KIT, CDH1, LSM7, C21orf4, DDI2 mRNA expression levels were significantly different between benign and malignant tumors, p(KIT) < 0.0001; p(CDH1) = 0.004; p(LSM7) = 0.03; p(C21orf4) = 0.01; p(DDI2) = 0.0001. No statistically significant difference was found for NATH, SYNGR2, TC1, Hs.296031. Among the markers, all but TC1 resulted expressed higher in benign samples compared to the malignant ones (Figure 1A).

### BRAF status

Among the 49 malignant samples, 28 carried the V600E mutation. All the benign samples were wild-type. Sensitivity and specificity of BRAF test were 57 and 100%, respectively.

### ROC analyses

We employed receiver-operated characteristics (ROC) curve analyses to determine model robustness for predicting malignancy in thyroid samples using the expression of each gene individually (Figure 1B, Table 1). Among the markers, KIT showed the highest AUC (0.9). We also performed a ROC analysis for the statistical significant markers (KIT, CDH1, LSM7, C21orf4, DDI2) and BRAF status in combination, the AUC resulted to be 0.8824, the sensitivity 91% and specificity 63% (Figure 1C). Although the AUC resulted quite similar to the KIT one, the predictive power increased when the markers were combined together.

**Table 1:** ROC analysis for each marker individually

	**Sensitivity**	**Specificity**	**AUC**^**a**^	**SE**^**b**^	**Thresholds Value**	**95% CI**^**c**^
KIT*	79.6	86.8	0,900	0.0313	≤ 0.105	0.817-0.954
CDH1*	61.2	73.7	0.700	0.0586	≤ 0.11	0.559-0.766
NATH	57.8	57.9	0.553	0.0658	≤ 0.112	0.440-0.662
LSM7*	69.4	57.9	0.625	0.0633	≤ 0.11	0.515-0.727
C21orf4*	58.3	73.7	0.644	0.0607	≤ 0.0001	0.533-0.744
DDI2*	56.2	86.8	0.729	0.0551	≤ 0.0026	0.622-0.819
SYNGR2	47.9	78.9	0.608	0.0613	≤ 0.04	0.497-0.712
TC1	85.0	38.2	0.581	0.0679	> 0.006	0.460-0.695
Hs.296031	77.8	32.4	0.490	0.0671	≤ 0.0051	0.375-0.605

^a^AUC (area under the curve).

^b^SE (standard error).

^c^CI (confidence interval).

*p < 0.05.

### Neural networks

The expression data of the markers were used to build Bayesian Neural Networks (BNN) in order to estimate the probability of thyroid malignancy.

We built several BNNs in order to find the most predictive one. This procedure uses a Probabilistic Neural Network (PNN) to classify cases into malignant and benign categories, based on 9 input variables (KIT, LSM7, C21orf4, DDI2, SYNGR2, TC1, Hs.296031, CDH1, NATH), by implementing a nonparametric method for classifying observations into one of benign and malignant groups based on the observed expression variables.

The Neural Network Bayesian Classifier made up of all markers has a predictive power of 80%, while the classifier made up of KIT, CDH1, LSM7, C21orf4, DDI2, TC1 and Hs.296031 resulted to have a predictive power of 87.7%.

The analysis was then conducted on 6 unknown samples. The pathological diagnosis for each sample was kept blinded until after the analysis was completed. When the blind was broken, we found that 5 of the 6 unknown samples were diagnosed by the model in concordance with the diagnosis determined by standard pathological criteria.

We also built a neural network classifier made up of the markers used in the most predictive model (KIT, CDH1, LSM7, C21orf4, DDI2, TC1 and Hs.296031) plus BRAF status. This classifier had a predictive power of 88.8%, and, more importantly, it resulted to completely discriminate the 6 unknown samples when the blind was broken (Table 2). When applying the BNN model, no classification errors came out when the probability of diagnosis was higher than 90%, thus allowing us to use this model as a correct predictor of samples with a probability score >90% (p < 0.0001).

**Table 2:** Probability values of the prediction model for the unknown samples

**Unknown samples**	**Benignity probability**	**Malignancy probability**	**Predicted diagnosis**	**Pathological diagnosis**
A	3.07E-07	1	Malignant	Malignant
B	0.294935	0.705065	Malignant	Malignant
C	0.427773	0.572227	Malignant	Malignant
D	7.09E-11	1	Malignant	Malignant
E	0.00012769	0.999872	Malignant	Malignant
F	0.94438	0.05562	Benign	Benign

### Role of molecular diagnosis in increasing the diagnostic accuracy of FNAC

We stratified the samples depending on either the histological and cytological diagnosis (Table 3) and then calculated the diagnostic gain obtained by applying BRAF molecular analysis, KIT expression model and BNN model to the indeterminate samples (Table 4).

**Table 3:** Histological and cytological diagnosis of 87 thyroid nodules

***Histological diagnosis***	***Cytological diagnosis***		
**PTC**^a^**: 49 cases**	**PTC**^a^	**SPTC**^b^	**IFP**^c^
	30 (61%)	14 (29%)	5 (10%)
**BN**^d^**: 38 cases**	**BN**^d^	**IFP**^c^	
	19 (50%)	19 (50%)	

^a^PTC: papillary thyroid carcinoma.

^b^SPTC: suspicious papillary thyroid carcinoma.

^c^IFP: indeterminate follicular proliferation.

^d^BN: benign.

**Table 4:** Role of molecular tests in the preoperative diagnosis

**CD**^**a**^	**BRAF V600E**	**KIT class 1 (malignancy probability 100%)**	**KIT class 4 (benignity probability 100%)**	**BNN**^**d**^**(probability score >90%)**
**SPTC**^b^	11(M^i^)	1(M^i^)		1(M^i^)
**IFP**^c^			3(B^h^)	8(B^h^)
	**CD**^e^	**MD**^f^ + **CD**^e^	**DG**^g^	
**correctly diagnosed samples**	49/87: 56%	73/87: 84%	+28%	

^a^CD: cytological diagnosis.

^b^SPTC: suspicious papillary thyroid carcinoma.

^c^IFP: indeterminate follicular proliferation.

^d^BNN: Bayesian neural network.

^e^CD: cytological diagnosis.

^f^MD: molecular diagnosis.

^g^DG: diagnostic gain.

^h^B: benign.

^i^M: malignant.

Among the indeterminate samples (IFP and SPTC) at the cytological level, 11 SPTC were correctly diagnosed as malignant by BRAF test, 4 additional samples were correctly classified by KIT model as 1 malignant and 3 benign, and 9 additional samples were diagnosed by the BNN model as 1 malignant and 8 benign. As shown in Table 4, when applying the molecular analysis, 13 malignant samples were moved to the diagnostic group of PTC and the total number of PTC raised from 30 (61%) to 43 (88%) with a malignancy diagnostic gain of 27%. Similarly, 11 IFP samples were moved to the diagnostic group of BN and the total number of BN rose from 19 (50%) to 30 (79%) with a benignity diagnostic gain of 29%.

Finally, if we consider both PTC and BN diagnoses, the whole diagnostic gain is of 28% with a statistically significant p-value of 0.0001.

### Correlation analysis

A multiple-variable analysis was performed to evaluate the correlation between the markers. The knowledge of the correlation of the markers could give us a better understanding of the mechanisms underlying thyroid cancer biology. In fact, the statistical correlation may reflect biologically correlation between markers.

**Figure 2:**
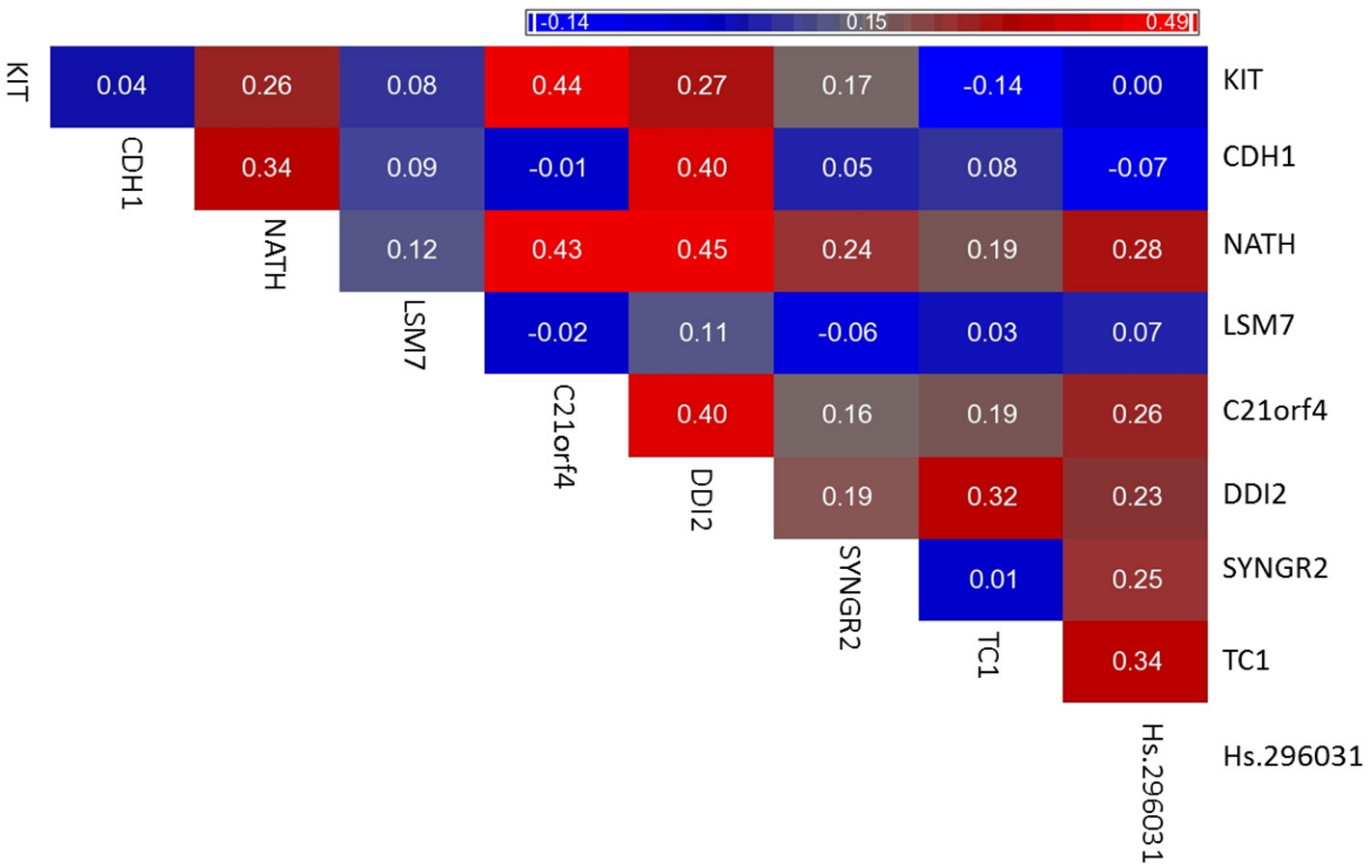
Similarity matrix of KIT, SYNGR2, C21orf4, Hs.296031, Hs.24183, CDH1, LSM7, TC1 and NATH based on Pearson’s correlation coefficient.

Pearson’s correlations between pairs of variable are reported in Figure 2.

## Discussion

Many candidate markers of thyroid cancer have been identified in microarray studies that require analytic and clinical validation in a cohort large enough to permit evaluation of their clinical utility. q-PCR has become a highly reliable technique that allows precise quantification of gene expression levels identified by microarray studies from various laboratories [20-22]. Moreover, q-PCR has been in clinical use as a diagnostic test in various fields of medicine.

Currently, the diagnosis of thyroid nodules relies primarily on cytology. For the majority of patients with PTC, FNA-based cytology can make a diagnosis with high accuracy [3]. However, there is a significant proportion of neoplasm in which the FNA-based preoperative cytological diagnosis fails.

The primary aim of this study was to find a diagnostic accurate preoperative assay able to distinguish benign from malignant thyroid neoplasm. We found 5 out of 9 proposed gene markers (KIT, LSM7, C21orf4, DDI2, CDH1) differentially expressed in malignant and benign thyroid samples with a significant p-value (<0.05).

Of particular interest is the down regulation of KIT and CDH1 in malignant samples.

We previously showed that the expression silencing of KIT gene is associated with the malignant phenotype of thyroid nodules and KIT expression may represent a useful tool in the preoperative management of thyroid lesions [7]. KIT is a well-known proto-oncogene. Other studies obtained findings similar to ours [6,23]. We speculated that in some cell types KIT expression positively regulates mitogenesis and is selected for in neoplastic transformation; in other tissues (such as thyroid tissue) KIT is involved in morphogenesis and differentiation and is, therefore, negatively selected in the course of tumor progression. Although the functional consequences of this modulation are unknown so far, KIT is likely to be relevant in regulating thyrocyte differentiation and survival, however further work is needed to elucidate the biological meaning of KIT down-expression in PTCs.

CDH1 encodes for E-cadherin. We found a down regulation of CDH1 expression in malignant samples and this is in perfect concordance with the literature. Loss of E-cadherin function or expression has been implicated in cancer progression and metastasis [24-26]. In fact, E-cadherin down-regulation decreases the strength of cellular adhesion within a tissue, resulting in an increase in cellular motility. This in turn may allow cancer cells to cross the basement membrane and invade surrounding tissues.

Regarding TC1, several studies reported a higher expression of this protein in thyroid malignancies compared to benign nodules. Concordant to the literature, we observed a tendency of TC1 to be overexpressed in our cohort of malignant samples, though not statistically significant. TC1 has been shown to interact with Chibby (Cby) [27], which regulates the β-catenin-mediated transcription antagonistically and thereby enhances the signaling pathway through relieving the suppression by Cby. TC1 regulation of Cby is of considerable biological significance in the Wnt/β-catenin pathway. Indeed TC1 up-regulates β-catenin target genes implicated in invasiveness and aggressive behaviour of cancer.

For the other markers it is difficult to speculate since their function and role in thyroid carcinogenesis are still largely unknown. Additional functional studies are needed to elucidate their role in thyroid cancer initiation and progression.

When assessing the diagnostic utility of the markers, KIT, LSM7, C21orf4, DDI2, and CDH1 had a high diagnostic accuracy. Thus, all the significant markers, alone and in combination, can be used to distinguish between malignant and benign FNA samples.

Recently, a new class of techniques known as Bayesian Neural Networks (BNN) have been used as a supplement or alternative to standard statistical techniques [11]. Since they do not require explicit distributional assumptions, BNNs have been employed for the classification of medical outcomes [11]. We developed a Bayesian Artificial Neural Network model based on data collected from FNA samples. Bayesian classification has been applied across the spectrum of medicine, from optimization of pharmacotherapy dosing [28,29], predicting cancer screening [30] and diagnostic test results [31,32], to determining injury severity [33], assessing operative risk [34] and predicting surgical outcomes [35-38]. We built several Neural Networks and the most predictive one has resulted to be made up of KIT, CDH1, LSM7, C21orf4, DDI2, TC1 and Hs.296031, with a power of 87.7%. The network was then validated on 6 unknown samples. The model determined the accurate diagnosis of 5 of 6 unknown samples tested, based on a comparison to the gold standard pathological diagnosis as determined by clinical pathologists.

It’s important to notice that we have put in the model also two non-significant markers (TC1, Hs.296031), because their contribution to the predictive power seemed to be relevant. In fact, some variables although not significant may increase the discriminative power to a model refining the predictions.

The classifier built using also BRAF mutational status resulted to have a predictive power of 88.8% and to successfully discriminate the unknown samples when the blind was broken (Table 2), thus the gene expression analysis combined to the BRAF mutational analysis may represent a very useful test to preoperatively discriminate benign from malignant thyroid tumors.

The probability of the prediction of diagnosis for almost all the samples resulted to range between 95% and 100%, thus, although the general prediction value is 88.8%, the predictive power to assess each sample individually can reach a value of 100%. These data also strengthen the importance of the 8-markers model as an adjunctive tool for the preoperative diagnosis of thyroid nodules.

We also stratified the samples depending on both the histological and cytological diagnoses (Table 3). The diagnostic gain obtained by applying BRAF molecular analysis, KIT expression model and BNN model was then calculated.

By applying the BNN model, no classification errors came out when the probability of diagnosis was higher than 90%, thus allowing us to use this model as a correct predictor of samples with a probability score >90% (p < 0.0001).

We then calculated the diagnostic gain after applying molecular tests (Table 4).

**Figure 3:**
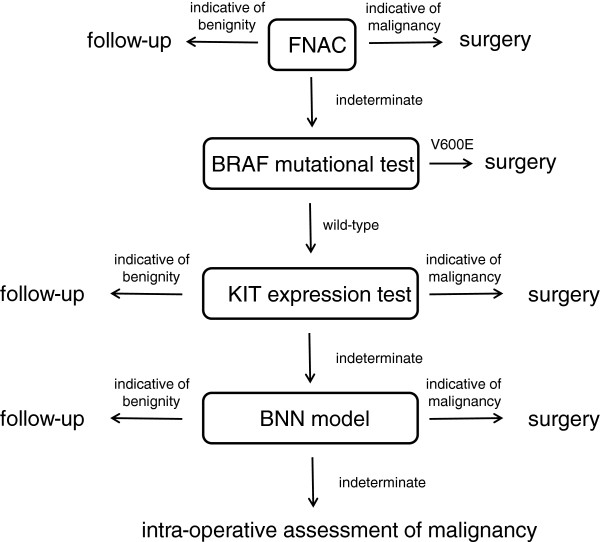
Diagram showing the preoperative assessment of thyroid malignancy.

Among the uncertain samples (IFP and SPTC) at the cytological level, 11 were correctly diagnosed by BRAF test, 4 additional samples by KIT model and 9 additional samples by the BNN model. It is important to point out that IFP lesions are often very difficult to diagnose even at frozen section and in this study we developed a molecular approach that is able to correctly classify as certain benign 46% (11/24) of IFP lesions. Therefore using molecular approaches these patients would have been clinically enrolled to the follow up group instead of sent to surgery. Thus, the combined use of the molecular tests resulted to produce a diagnostic gain of 28% (Table 4). Basically, what we propose in this paper is the use of BRAF molecular analysis (after uncertain cytological diagnosis) to assess the malignancy of thyroid nodules in the first place, then the use of KIT model for the indeterminate nodules and at last the use of the 8-gene model to ultimately assess the diagnosis of the nodules that otherwise would remain suspicious (Figure 3). The combinatorial power of these tools could definitely increase the percentage of thyroid nodules correctly classified while decreasing the ones remained indeterminate.

All these findings strengthen the importance of molecular pathology where morphology and molecular alterations represent a powerful approach to diagnosis. In this line, this study aimed to assess the diagnostic potential of the 8-gene expression model as an adjunctive tool in the preoperative management of thyroid nodules. We demonstrated that the 8-gene expression model provides an increased diagnostic power to the molecular pathology approach based on BRAF mutation and KIT expression analysis.

We also performed a multiple variable analysis among all the markers, independently on the diagnostic classification, in order to evaluate a possible functional correlation among the markers (Figure 2). In literature there is no evidence about the biological correlation among the well-studied markers; however it is interesting to note that the unknown marker Hs.296031 statistically correlates with NATH, C21orf4, DDI2, SYNGR2 and TC1. This may reflect also a biological correlation, thus, further studies are needed to explore this phenomenon.

## Conclusion

The genetic classification obtained with the model here presented is highly accurate and may provide a tool to overcome the difficulties in today’s preoperative diagnosis of thyroid malignancies. We hoped that the quantitative nature of this test will be a useful gene-based objective adjunct to the preoperative diagnosis of a disease that currently relies solely on cytology.

## Competing interests

The authors declare that they have no competing interests.

## Authors’ contributions

ST carried out the study, analysed the data and wrote the manuscript draft. CM and GB conceived of the manuscript, participated in its design, coordination, analysis and interpretation of data and supervised the writing of the manuscript. PA participated in the statistical data analysis. All the authors made intellectual contributions and approved the final manuscript.

## Pre-publication history

The pre-publication history for this paper can be accessed here:

http://www.biomedcentral.com/1471-2407/12/396/prepub
